# A controlled synthesis method of alkyl methacrylate block copolymers *via* living anionic polymerization at ambient temperature

**DOI:** 10.1039/c9ra01577a

**Published:** 2019-05-22

**Authors:** Zheng Li, Jianding Chen, Guijin Zou, Tongyuan Zhang, Dafu Wei, Xiang Xu, Yong Guan, Anna Zheng

**Affiliations:** Key Laboratory of Special Functional Polymeric Materials and Related Technology of the Ministry of Education, School of Materials Science and Engineering, East China University of Science and Technology Shanghai 200237 P. R. China yguan@ecust.edu.cn zan@ecust.edu.cn

## Abstract

A controlled synthesis method of alkyl methacrylate block copolymers such as poly(methyl methacrylate)-*b*-poly(ethyl methacrylate) (PMMA-*b*-PEMA), poly(methyl methacrylate)-*b*-poly(butyl methacrylate) (PMMA-*b*-PBMA) and poly(ethyl methacrylate)-*b*-poly(butyl methacrylate) (PEMA-*b*-PBMA) *via* living anionic polymerization was innovated with potassium *tert*-butoxide (*t*-BuOK) as initiator in tetrahydrofuran(THF) solvent. The sequential anionic copolymerization could be smoothly conducted at 0 °C and the conversion of all monomers reached up to almost 100%. The copolymers were characterized by gel permeation chromatography (GPC), proton nuclear magnetic resonance (^1^H-NMR), fourier transform infrared spectroscopy (FTIR) and dynamic mechanical analysis (DMA). It was found that all block copolymers were in a narrow MWD while *M*_w_ and weight ratio of each block were coincided with the theoretical values and feed ratio. DMA measurement indicated that all the block copolymers have two glass transition temperatures which have proved the certain microphase separation and the partial compatibility of the blocks. The similar results were achieved after changing feed order or addition amount. Furthermore, the reactivity ratio was also studied and confirmed that reactivity ratio of MMA was the largest among alkyl methacrylate. Based on these results, the anionic block copolymerization containing polar alkyl methacrylate monomers at a commercial scale starts to become possible.

## Abstract 2

A series of well-defined diblock copolymers containing PMMA, PEMA and PBMA blocks were synthesized with narrow MWDs and high yields *via* anionic polymerization, while *t*-BuOK was selected as initiator in THF at 0 °C. .

## Graphical abstract

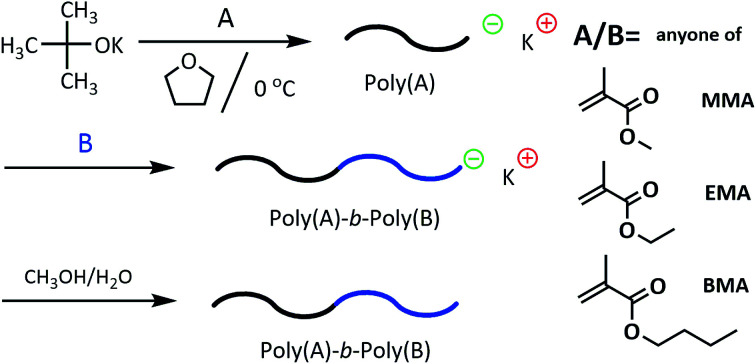

## Introduction

Research on living anionic polymerization has never been interrupted since the polymerization mechanism was reported by Scwarc in 1956^1–4^ due to its outstanding advantages such as the narrow distribution, controllable molecular weight and definite molecule structures. Anionic polymerization has been recognized as the most suitable method to prepare homopolymers and block copolymers with high purity and well-defined structures.^5–8^ Decades of researches have realized the application of anionic polymerization in the industry, and block copolymers such as polystyrene-*b*-polyisoprene/polybutadiene-*b*-polystyrene (SIS/SBS) have entered the market which were utilized in the adhesive, plastic modification and so on.^9–11^ However, up to this day, there are few copolymers contained polar monomer blocks such as alkyl methacrylate synthesized by anionic polymerization in industrial scale for much side reactions would occur in the anionic polymerization at ambient temperatures and some initiator system might be complex or expensive for industrialization.^12^

Indeed, because of no double bonds in the main chain, the copolymers contained alkyl methacrylate monomer blocks would have excellent properties oil, oxidation and aging resistance. On the other hand, glass transition temperatures of homopolymers would change along with different alkyl methacrylate monomers from −60 °C (laurate methacrylate (LMA)) to 110 °C (isobornyl methacrylate (IBOMA)).^13^ Copolymer blocks would lead to micro-phase separation and show excellent mechanical properties,^14^ thus, these types of novel block copolymers would have wide application prospect. In order to control side reactions and structures of polar monomers, low temperatures and some inhibiting ligand have been adopted for anionic polymerization in recent years.^15,16^ Copolymers contained PMMA blocks such as polyethylene-*b*-polypropylene-*b*-PMMA (PE-*b*-PP-*b*-PMMA) and PE-*b*-polyhexene-*b*-PMMA (PE-*b*-PH-*b*-PMMA) have been obtained *via* anionic polymerization while fluorenyl-amide ligated titanium/methylaluminoxane/2,6-di-*tert*-butyl-4-methylphenol was used as complex initiating system.^17^ Poly(1-adamantyl acrylate) (PAdA) and PAdA-*b*-PMMA block copolymers of narrow MWDs (1.10) were synthesized successfully in THF solvent at −78 °C with *sec*-butyl-lithium (*s*-BuLi)/1, 1-diphenylethyl (DPE)/lithium chloride (LiCl), diphenylmethyl potassium (DPMK)/diethyl zinc (Et_2_Zn) or sodium naphthalenide (Na-Naph)/DPE/Et_2_Zn as initiator respectively.^18^ Synthesis of poly(methyl methacrylate)-*b*-poly methyl (3,3,3-trifluoropropyl) siloxane (PMMA-*b*-PMTFPS) diblock copolymers was conducted *via* anionic polymerization initiated by alkyl lithium functionalized by acetal.^19^ A plug flow reactor was chosen as reaction vessel to synthesize PS-*b*-PMMA with high molecular weight in THF at −78 °C.^20^ PS-*b*-PI-*b*-PMMA tri-block copolymer was also produced in which PS-*b*-PI di-block copolymer polymerized in toluene at room temperature firstly and then the anionic polymerization of MMA was carried out at −78 °C after LiCl/THF adding to the system.^21^ Moreover, anionic polymerization of dendrimer-like star-branched poly(*tert*-butyl methacrylate)s (P*t*BMA) and their block copolymers were also studied at −78 °C.^22^ However, the polymerization was conducted at low temperatures or initiated by complex and expensive compound which could not be realized in industrialized conditions, which limited the further application.

Our research group has made great efforts on anionic polymerization of polar monomers to explore chain initiation and inhibit side reactions at ambient temperature. A compound so called “P-Complex”^23^ was synthesized by our group and used as an effective inhibitor to control the side reactions in the anionic polymerization of MMA and its copolymers. Followed the research above, PI-*b*-PMMA and PBMA-*b*-PMMA with about 1.2 of MWD were obtained successfully at 0 °C when using *n*-butyllithium (*n*-BuLi) as initiator and “P-Complex” as inhibitor.^24^ The triblock copolymer PS-*b*-PI-*b*-PMMA^25^ was gained and the anionic polymerization was conducted in hydrocarbon (CH) solvent with trace amounts of THF as a polar regulator. Especially, the reaction temperature of MMA was elevated to 0 °C. The structure of PS-*b*-PI-*b*-PMMA was compared with predicted and showed obvious microphase separation according to measurement. A counter ion exchange initiating system potassium *tert*-butoxide (*t*-BuOK)/*n*-BuLi^26^ was researched for anionic polymerization of alkyl methacrylate. Then block copolymer PMMA-*b*-P*t*BMA was achieved in THF solvent at the temperatures ranging from 0 °C to 40 °C. It has been shown that *t*-BuOK was an effective initiator for anionic polymerization of alkyl methacrylate^27,28^ but whatever it was controlled for anionic polymerization has not ever deeply researched.

Therefore, a series of copolymers containing PMMA, PEMA and PBMA blocks were prepared *via* anionic polymerization with *t*-BuOK as initiator in THF solvent at ambient temperature. The conversions of monomers were also focused. GPC, ^1^H-NMR and DMA measurement were used to investigate the *M*_w_ and the structures of gained copolymers. These results were summarized and compared with the theoretical values to confirm this initiating system would be a controlled method for alkyl methacrylate block copolymers. Furthermore, the reactivity ratio measurement of alkyl methacrylate was also carried out and discussed.

## Experimental

### Materials

Methyl methacrylate (MMA, AR, Shanghai Macklin Biochemical Co., Ltd.), ethyl methacrylate (EMA, AR, Shanghai Macklin Biochemical Co., Ltd.) and butyl methacrylate (BMA, AR, Shanghai Lingfeng Chemical Reagent Co., Ltd., China) were purified by distillation in a vacuum system after stirring with calcium hydride (CaH_2_, Shanghai Titan Scientific Co., Ltd.) for 48 hours, and soaked with 4 Å molecular sieves at −20 °C temperature for more than 24 hours before use. Tetrahydrofuran (THF, AR, Shanghai Titan Scientific Co., Ltd.) as solvent was refluxed with sodium (Na, Shanghai Lingfeng Chemical Reagent Co., Ltd., China) at certain temperatures for 48 h, distilled and soaked with 4 Å molecular sieves. Potassium *tert*-butoxide (*t*-BuOK, 99%, Sinopharm Chemical Reagent Co., Ltd., China) was used as received. Argon (Ar, 99.99999%, Air Liquid Shanghai Co., Ltd) was purified by flowing through two connected cylinders filled with 4 Å molecular sieves. Methanol (CH_3_OH, Shanghai Titan Scientific Co., Ltd.) was used as received.

### Characterization methods

The molecular weight and its distribution (MWD) were determined by multi-detector gel permeation chromatography (Water 1515 system; Waters corporation, America), equipped with an 18 angles laser scattering detector (LS signal) and a refractive detector (RI signal), using THF as the eluent at a flow rate of 1.0 ml min^−1^ at 25 °C.

^1^H-NMR spectra was measured by a BRUKER AV400 spectrometer with CDCl_3_ as solvent and tetramethyl silane (TMS) as the internal reference.

Fourier transform infrared spectroscopy (FTIR) was measured by Nicolet 6700 spectrometer over the wave number range of 4000–400 cm^−1^.

Dynamic mechanical spectrometer (Q800, TA Co., USA) was used to study the dynamic mechanical properties of the copolymers. The samples were transited into high elastic state by raising temperature and then poured into rectangular strips with size of 30 mm × 12 mm × 3 mm, tested with single cantilever bending mode and heated from −10 to 125 °C, at heating rate of 10 °C min^−1^ and frequency of 0.5 Hz in a nitrogen atmosphere.

Determination of monomer reactivity ratios was carried out by using the extended Kelen–Tüdos method.

### Synthesis of homo polymers

All the polymerization and reactions were carried out under Ar atmosphere and all the flasks were inflated by Ar after baked. The initiator solution (0.060 mol L^−1^) was prepared by adding a certain amount of *t*-BuOK (0.34 g) and THF (50 ml) as solvent in a three-necked flask under Ar atmosphere. The solution was then cooled to the 0 °C and kept for 30 min. The anionic homopolymerization of alkyl methacrylate monomers (MMA/EMA/BMA) was performed at 0 °C with *t*-BuOK as initiator. The product was quenched by degassed CH_3_OH and the solution was concentrated by rotary evaporator before being precipitated into an excess of 90/10 (v/v) CH_3_OH/H_2_O mixture under stirring. The crude product was vacuum dried at 120 °C for 12 hours.

### Synthesis of block copolymers

The glass reactors and initiator solution were treated in the same way as that of homo polymerization. The solution was then cooled to the 0 °C and kept for 30 min, followed by addition of the first monomers. After a certain amount of time, the second set of monomers were added. Post-treatment of the copolymer obtained was the same as above.

## Results and discussion

### Synthesis and characterization of homopolymers

As our previous study^27,28^ showed, *t*-BuOK was capable of initiation for anionic polymerization of alkyl methacrylate at ambient temperature. For convincing this synthesis method, another homopolymerization of MMA, EMA and BMA was conducted and the results were as Table 1 showed. According to previous study, the concentration of *t*-BuOK solution was chosen as 0.060 mol L^−1^ with THF as solvent at 0 °C.

**Table 1:** Anionic homopolymers of MMA, EMA and BMA^a^

#	Polymer	*M*_n_ (×10^−3^)	MWD	Yield (%)	Eff.^b^ (%)
1	PMMA	3.31	1.22	99.40	45.65
2	PEMA	4.67	1.18	98.38	43.03
3	PBMA	5.04	1.14	98.79	42.25

a *V*_THF_ = 50 ml, *C*_*t*-BuOK_ = 0.060 mol L^−1^, *C*_monomer_ = 0.942 mol L^−1^.

b Initiator efficiency (I*) = *M*_n(calcd)_/*M*_n(obsed)_, where *M*_n(calcd)_ = MW_(monomer)_ × [monomer]/[initiator] × conversion% + MW of chain end groups.

The PMMA, PEMA and PBMA were synthesized *via* anionic polymerization with *t*-BuOK as initiator. The GPC results confirmed the polymers were kept in a narrow MWD and high yields. Obviously, for the existence of association state in *t*-BuOK solution, the initiator efficiency was not close to 100% but at a stable number of approximately 43% at this situation, which has been discussed in our previous work.^27,28^ This consequence would provide a possibility of a controlled method for block copolymers of alkyl methacrylate.

**Fig. 1:**
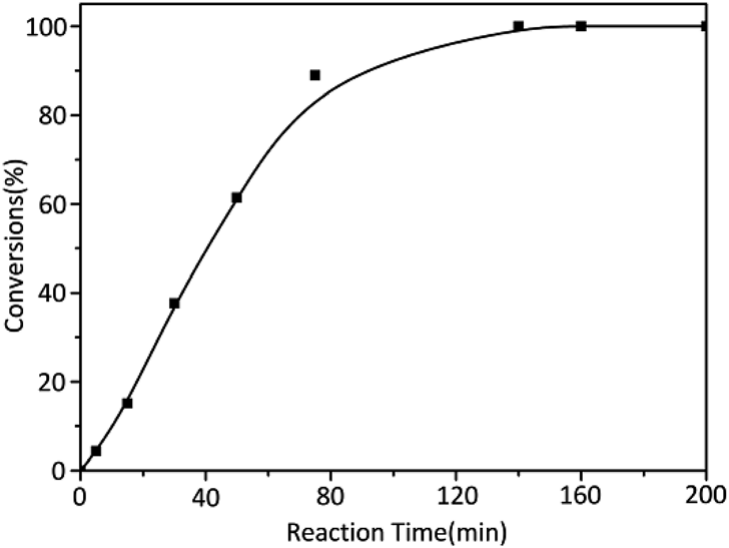
Anionic polymerization kinetics of MMA initiated by *t*-BuOK.

On the other hand, in order to determine the reaction time of each block polymer, the kinetic of alkyl methacrylate monomers was studied with *t*-BuOK as initiator. The results of reaction time were summarized in Table 2 while three monomers were of the same molar amount. The reaction rate of MMA was obviously slow where the conversion of MMA was nearly 100% until about 150 min (Fig. 1). Considering the reaction temperature was at ambient temperature and yields were almost complete, a grand development has been achieved to the previous study.

**Table 2:** Anionic homopolymerization reaction time of MMA, EMA and BMA^a^

#	Monomer	*m*_*t*-BuOK_ (g)	*C*_*t*-BuOK_ (mol L^−1^)	Conversion at 35 s (%)	End time (s)
a	MMA	0.345	0.061	1.10	9000
b	EMA	0.340	0.060	78.60	108
c	BMA	0.339	0.060	83.33	96

a *C*_MMA_ = *C*_EMA_ = *C*_BMA_ = 0.942 mol L^−1^.

**Fig. 2:**
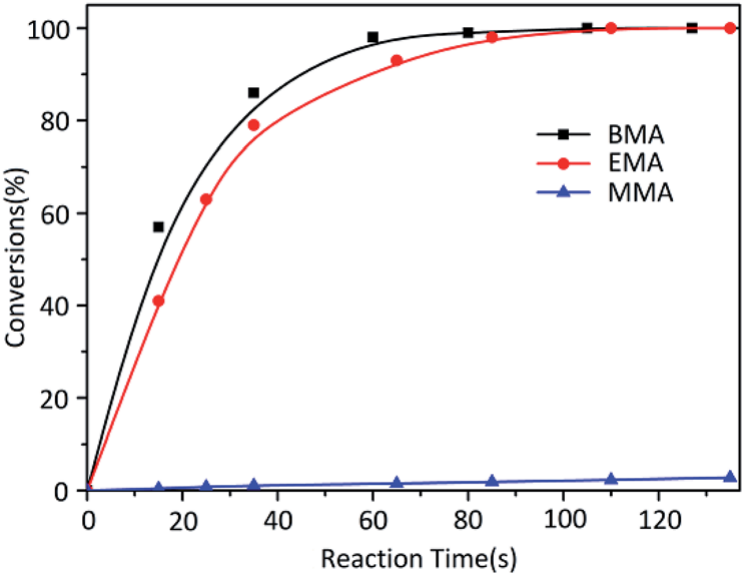
Anionic polymerization kinetics of MMA, EMA and BMA initiated by *t*-BuOK.

While the reactions were terminated at 35 s by CH_3_OH, the conversion of each monomer was measured. Contrary to the result of MMA, other two monomers EMA and BMA had quite faster reaction rates (Fig. 2). According to classical theory, MMA has the shortest carbon chain length and largest polarity among three monomers so that MMA should have the fastest reaction. Reaction rate would decrease in the order of carbon chain length; however, our research results were poles apart from the prediction. At the same time, in the previous study,^26^ the reaction rate of *t*-BMA was ten times faster than that of MMA when the polymerization was initiated by *n*-BuLi/*t*-BuOK initiating system, as a collateral evidence.

As we all known, K^+^ as the counter ion would had positive electrical property reduce a lot instead of Li^+^ controlling the distance much bigger between ion pairs. On the other hand, comparison among three monomers' size obviously MMA < EMA < BMA. According to the “Ion Pair Channel Idea”,^29^ the required space for MMA to insert into was least, nevertheless, the difference between channel space of ion pairs and MMA's required space was much huge, causing the reaction rate of MMA the slowest. The required space increased in the order of EMA and BMA which grown closer to the distance between K^+^ and anion, so it's reasonable that reaction rate become faster.

According to the results above, the reaction time of each block polymer was determined: 3 h for the PMMA block while 0.1 h for the PEMA and PBMA blocks.

### Synthesis and characterization of block copolymers

Based on the results above, the block polymerization contained PMMA, PEMA and PBMA blocks was conducted at the same reaction condition. The results of block polymers were summarized in Table 3.

**Table 3:** Anionic block polymerization contained PMMA, PEMA and PBMA blocks

#	Polymer	*C*_1_ (mol L^−1^)	*C*_2_ (mol L^−1^)	Time (h)	*M*_n_ (×10^−3^)	MWD	Yield (%)	Wt_n1_ : Wt_n2_	
								Calcd	Obsed^a^
4	PMMA-*b*-PEMA	0.941	0.942	3 + 0.1	7.32	1.22	98.5	46.7 : 53.3	46.2 : 53.8
5	PEMA-*b*-PMMA	0.942	0.943	0.1 + 3	7.41	1.26	97.6	53.3 : 46.7	53.3 : 46.7
6	PMMA-*b*-PEMA	0.941	1.885	3 + 0.1	9.78	1.28	99.0	63.1 : 36.9	64.1 : 35.9
7	PMMA-*b*-PBMA	0.942	0.942	3 + 0.1	8.48	1.33	97.8	42.0 : 58.0	40.8 : 59.2
8	PBMA-*b*-PMMA	0.942	0.943	0.1 + 3	8.12	1.28	98.4	58.7 : 41.3	57.5 : 42.5
9	PMMA-*b*-PBMA	0.943	1.884	3 + 0.1	11.05	1.21	98.1	58.5 : 41.5	57.9 : 42.1
10	PEMA-*b*-PBMA	0.942	0.943	0.1 + 0.1	10.93	1.26	99.2	44.5 : 55.5	45.0 : 55.0
11	PEMA-*b*-PBMA	0.942	1.882	0.1 + 0.1	14.72	1.36	97.5	61.0 : 39.0	61.9 : 38.1

a Calculated by ^1^H-NMR.

**Fig. 3:**
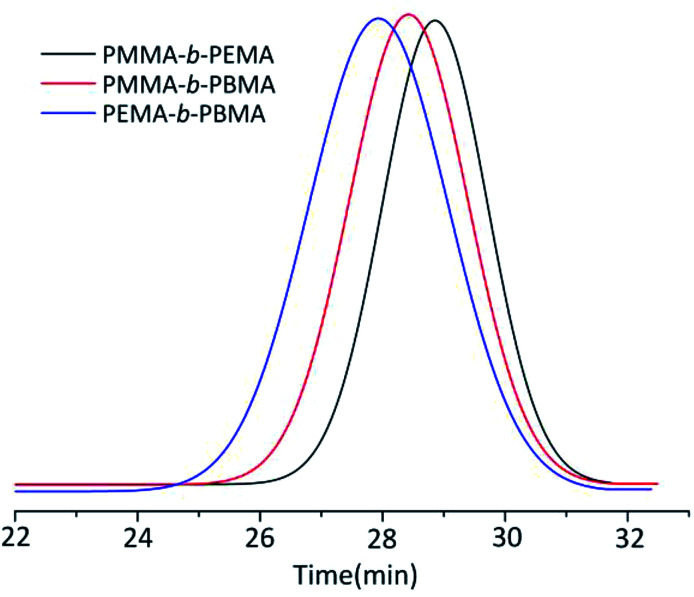
GPC curves of three block copolymers (sample 4, 7 & 10).

The MWD of block copolymers extended to 1.25–1.30 which was slightly larger than that of homopolymers. Considering there were no ligand and the temperature was at 0 °C, the MWD of block copolymers was rather narrower than other copolymers synthesized by different method.^3^ Taking the sample 4, 7 & 10 GPC curves as examples (Fig. 3), the *M*_n_ of copolymers from GPC results was rather close to the calculated ones (the initiator efficiency of *t*-BuOK was set as 43.5% at this condition) and the yield still reached up to 100%, which was a great breakthrough for anionic polymerization of MMA and other alkyl methacrylate. The GPC results showed a significant single peak distribution and the peaks moved to the left of shorter elution time along with the larger molecule weight. This phenomenon strongly confirmed the copolymers were strict block copolymers.

**Fig. 4:**
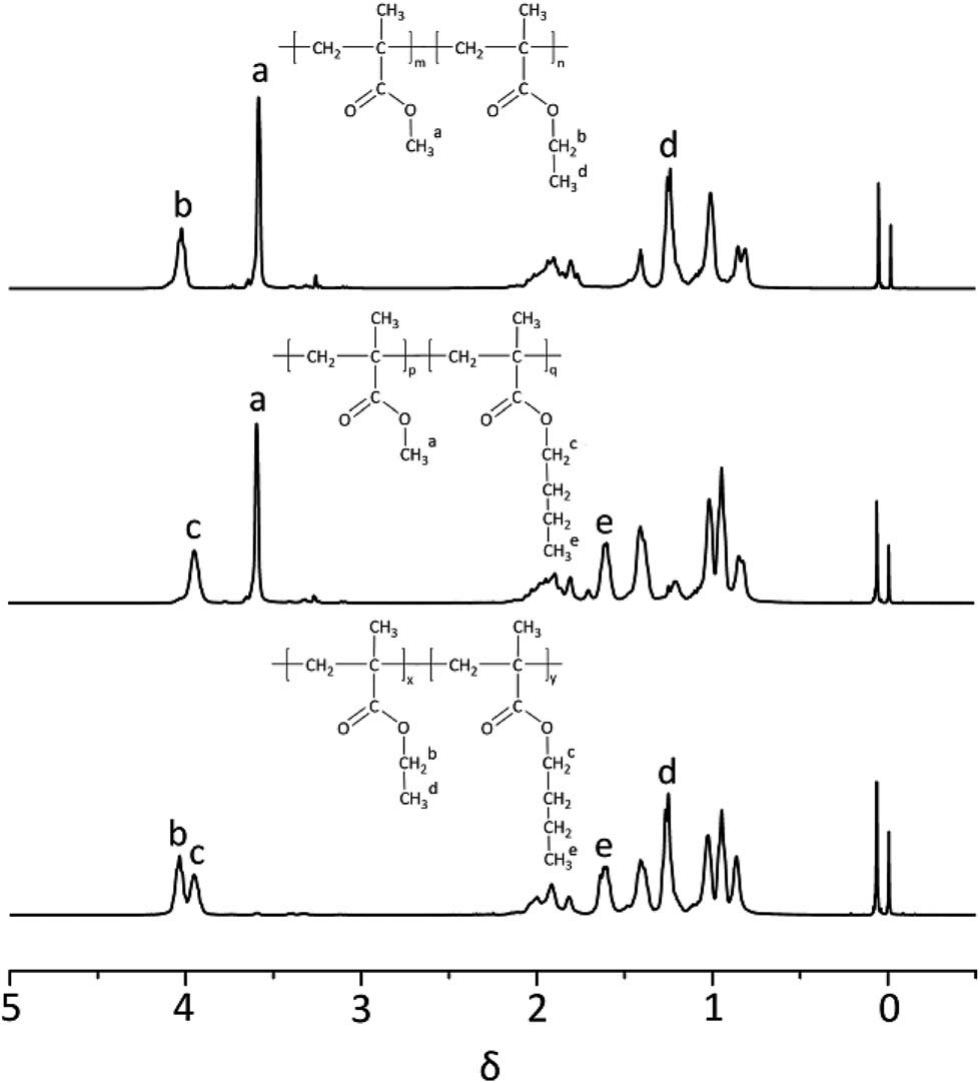
^1^H-NMR spectrum of three block copolymers (sample 4, 7 & 10).

^1^H-NMR spectrum of three block copolymers such as PMMA-*b*-PEMA (sample 4), PMMA-*b*-PBMA (sample 7) and PEMA-*b*-PBMA (sample 10) were revealed as typical examples to analyze the structures of block copolymers in Fig. 4. Some peaks at *δ* 3.58 (a) assigned to protons of methoxy group (–OCH_3_) in PMMA blocks. Furthermore, a signal at *δ* 4.02 (b) attributed to protons of methylene connected with the ester group (–OCH_2_–) and the signal at *δ* 1.25 (d) corresponded to the protons of the methyl at the end of side chain (–CH_3_) in PEMA blocks. Besides, there were a peak at *δ* 3.96 (c) attributing to protons of methylene connected with the ester group (–OCH_2_–) and a signal at *δ* 1.60 (e) belonged to the second methylene at the side chain (–CH_2_–) in PBMA blocks.

Calculating the ratio of the integral areas of these characteristic peaks, the mole ratio of each block could be figured out as showed in Table 3. For example, in sample 4, the mole ratio of MMA and EMA was 1 : 1.02, which was approximate to the theoretical value (1 : 1). Naturally, the weight ratio of PMMA and PEMA was worked out 46.2 : 53.8, closed to the feed ratio (46.7 : 53.3). Expectedly, the weight ratios of other samples approached to the feed ratios while feed order or additive amount was changed. These results proved quietly little side reaction existed during the anionic polymerization of alkyl methacrylate and the conversions of each block were completed. The block copolymers of alkyl methacrylate monomers were synthesized successfully in a controlled method with *t*-BuOK as initiator which the final products accorded with the theoretical calculation.

Compared sample 4 & 6, 7 & 9 and 10 & 11 while all the later samples doubled the addition of the first block, the observed *M*_n_ increased with the addition of monomers were closed to the calculated. After exchanging the addition order of monomers with the same concentration, all reactions were nearly completed and the *M*_n_ almost unchanged. These any anions of three kinds of alkyl methacrylate monomers (MMA, EMA & BMA) could initiate each other. Whatever the concentration or order of monomers was changed, the copolymers could be always gained with narrow MWDs, high yields and *M*_n_ which could be designed.

**Fig. 5:**
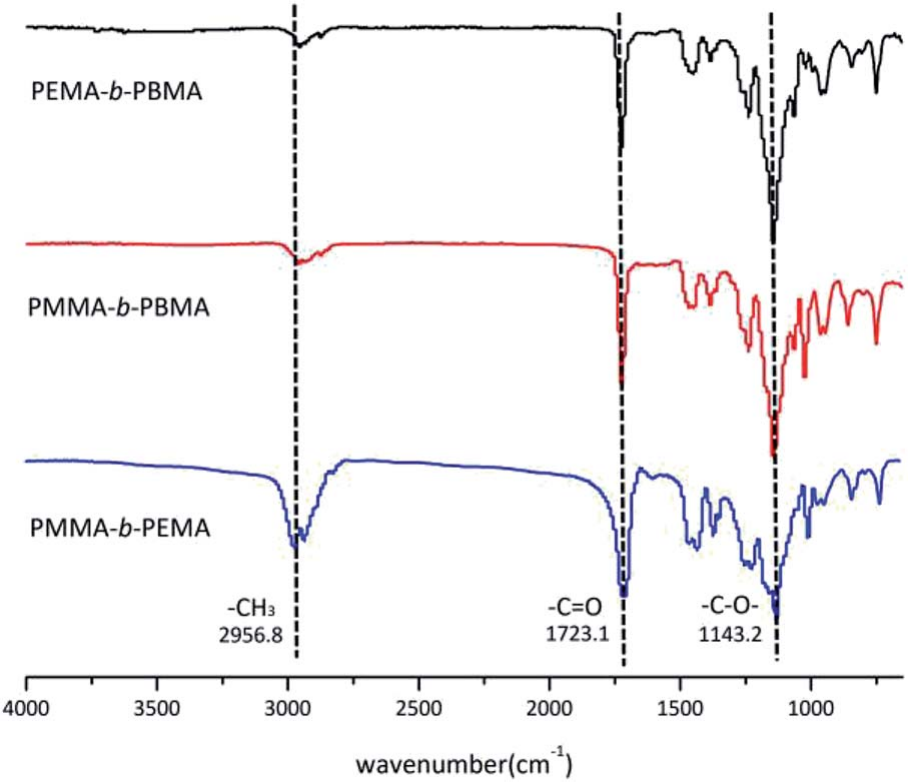
FTIR spectra of three block copolymers (sample 4, 7 & 10).

The structure of block copolymers was confirmed by FTIR and Fig. 5 showed the FTIR spectra of sample 4, 7 & 10. Because three copolymers were almost same in structure except the side chain, there were several common peaks in spectra. The absorption bands at 2956.8 cm^−1^ belonged to methyl (–CH_3_) vibration in main chains. The bands at 1723.1 cm^−1^ and 1143.2 cm^−1^ resulted from the carbonyl (C=O) and –C–O– stretching vibration of ester group in blocks. On the other hand, there was no peak at 3185 cm^−1^ which attributed to *tert*-butyl (–C(CH_3_)_3_) of *t*-BuOK.^30^ This phenomenon mean that few initiator remained in the copolymers after treatment.

**Fig. 6:**
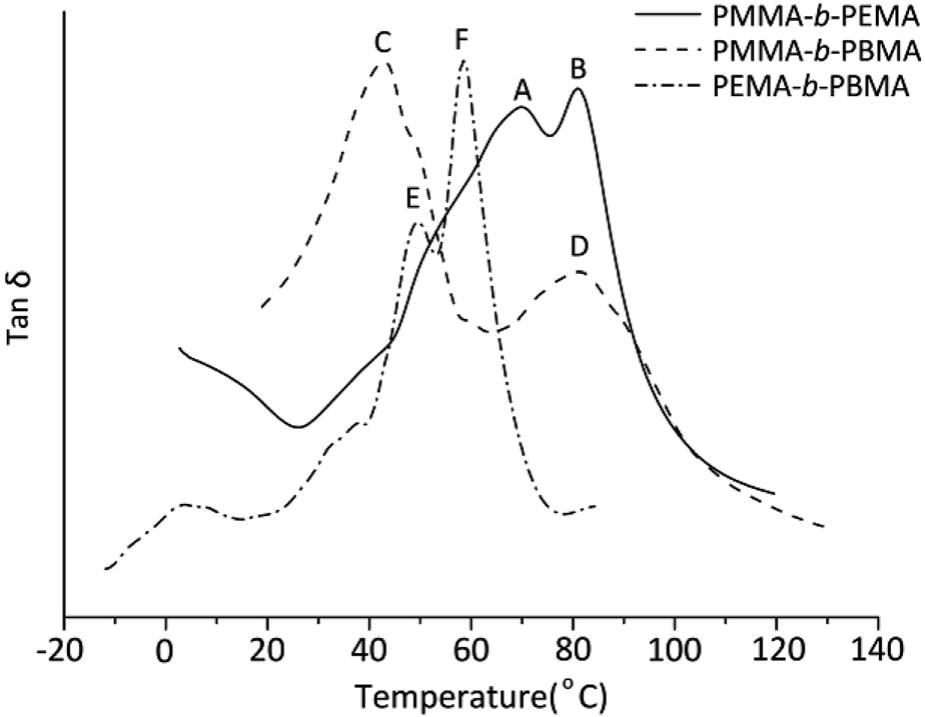
Dynamic mechanical curves (tan *δ*) of three block copolymers (sample 4, 7 & 10).

To analyze the dynamic mechanical behavior of block copolymers, the glass transitions of sample 4, 7 & 10 were measured by DMA. For indicating the glass transitions temperatures clearly, the three curves of loss tangent were plotted in the same figure (Fig. 6). Obviously, in any copolymers, there are two glass transitions which confirmed the copolymers synthesized were definite block copolymers. In the PMMA-*b*-PEMA copolymer, there are two peaks in tan *δ* curves, where peak A belongs to PEMA blocks at 70.1 °C and peak B at 83.1 °C indicated the *T*_g_ of PMMA blocks. Similarity, peak C (41.2 °C) was attributable to *T*_g_ of PBMA blocks and peak D (82.4 °C) was assigned to PMMA blocks in PMMA-*b*-PBMA. Especially, there are two close peaks in the DMA curves of PEMA-*b*-PBMA where peak E belongs to PBMA blocks at 49.2 °C and the temperature at peak F (59.2 °C) was *T*_g_ of PEMA blocks.

Indeed, on the basis of our GPC and ^1^H-NMR results above,the *M*_w_ of PMMA, PEMA and PBMA blocks in three copolymers would be nearby, however, the glass transition temperatures of same blocks in different copolymers have a certain difference. For a more intuitive presentation of comparison, the data was listed in Table 4. Taking PMMA blocks as example, according to literature and book,^31^ after calculated using eqn (1):1
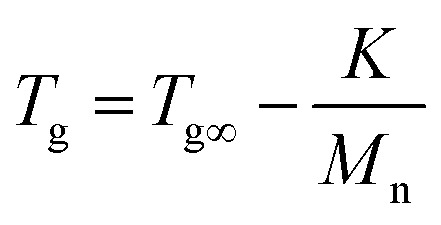
where *T*_g_ is the glass transition temperature of the polymers while *n* being high enough and the *K* is the effect parameter of the *T*_g_ on *n*. The theoretical *T*_g_ was about 95 °C but two observed *T*_g_ are both lower than 95 °C at approximate 80 °C. Moreover, the theoretical *T*_g_ of PBMA blocks was calculated out about 21 °C but the two actual *T*_g_s are observed at more than 40 °C which are higher than theoretical temperatures. In particular, the experimental *T*_g_ of PEMA blocks are on the either side of calculated *T*_g_ at about 65 °C which are different from those of PMMA and PEMA blocks. According to the polymer physic, this phenomenon may be due to the partial compatibility of blocks for the relatively low *M*_w_ of each block (less than 6000) and the similarity structures. The compatibility would lead to the glass transition temperatures of one blocks in block copolymers shift to the glass transition temperatures of another blocks. These results not only clearly demonstrated the micro-phase separation existing in the block copolymers but also suggested that there was partial compatibility of the block molecular chains in the copolymers.

**Table 4:** Observed and calculated glass transition temperatures of PMMA, PEMA & PBMA blocks

		PMMA	PEMA	PBMA
*T*_g_ (obs) (°C)	PMMA-*b*-PEMA	83.1	70.1	—
	PMMA-*b*-PBMA	82.4	—	41.2
	PEMA-*b*-PBMA	—	59.2	49.2
*T*_g_ (cal) (°C)		∼95	∼65	∼21

All the results above confirmed that a series of well-defined diblock copolymers were synthesized successfully. Considering the temperature was at 0 °C and *t*-BuOK was used as single initiator without any ligand, this method would have potential of industrialization.

### Reactivity ratio measurement of alkyl methacrylate

Among several methods which are available to determine a monomer reactivity ratio, the Finemann–Ross, Ua Yezrielev–Brokhina–Roskin, and Kelen–Tüdós methods are appropriate at low conversions. The Mayo–Lewis and extended Kelen–Tüdós methods consider the drift in the comonomer and copolymer compositions with conversion. In that respect, it has been found that the most reliable method is the extended Kelen–Tüdós one, since one may simply use a linear graphic technique to calculate the reactivity ratio values with a very small error until the conversion is up to 60%.^32^

In addition, for the present living anionic copolymerization, only data at relatively high conversion could be obtained due to the very high reaction rate. The extended Kelen–Tüdós method was thus used to determine the monomer reactivity ratios.

Controlling the conversions between 15% and 30% and different molar ratio, a series of copolymers with different units were gained for reactivity ratio measurement. Moreover, the extended Kelen–Tüdós method is expressed by the following equations:2
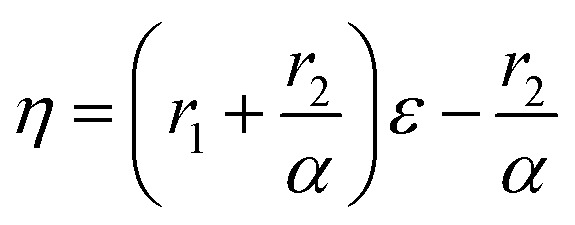
3
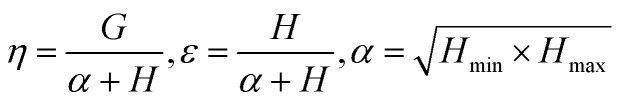
4
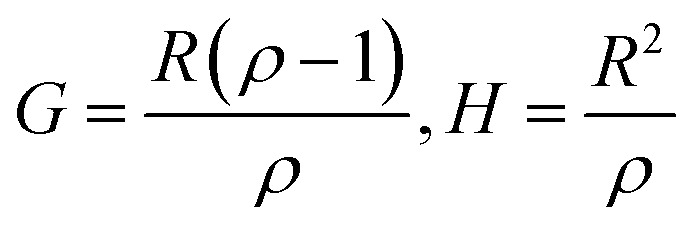
where *R* and *ρ* represent the molar ratios of monomer 1 to monomer 2 in the comonomer feed and the resulting copolymer, while *r*_1_ and *r*_2_ stands for the reactivity ratio of monomer 1 and monomer 2.

**Fig. 7:**
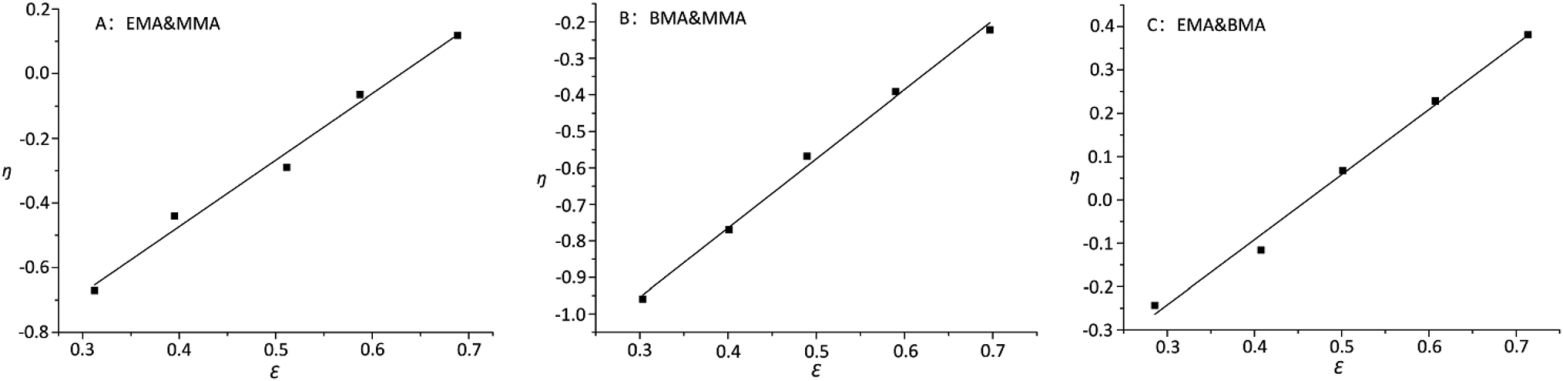
Extended Kelen–Tüdós plot ((A) EMA & MMA; (B) BMA & MMA; (C) EMA & BMA).

On the other hand, it was observed that –OCH_2_– and –OCH_3_ resonances do not split while varying the composition of copolymer PMMA-*c*-PEMA, leading to sharp peaks at 4.02 (b) and 3.58 (a), respectively. Therefore, the composition of copolymer samples was determined by direct integration of these two signals and the results of Kelen–Tüdós parameters were summarized in Table 5. While the *α* was calculated as 1.323, the linear regression equation is ‘*η* = 2.05*ε* − 1.29’ (Fig. 7) and the intercepts at *ε* = 0 and *ε* = 1 of the *ε versus η* plot gives *r*_EMA_ = 0.76 and *r*_MMA_ = 1.71, respectively.

**Table 5:** Extended Kelen–Tüdós parameters for anionic copolymerization of mixtures of MMA/EMA, MMA/BMA and EMA/BMA

No.	m1 & m2	*V*_m1_ (ml)	*V*_m2_ (ml)	*R*	*ρ*	*G*	*H*	*η*	*ε*	*α*
1	EMA & MMA	7	3	1.978	1.342	0.502	2.919	0.118	0.689	1.323
2		6	4	1.271	0.865	−0.207	1.880	−0.065	0.587	
3		5	5	0.848	0.521	−0.782	1.382	−0.290	0.511	
4		4	6	0.565	0.374	−0.962	0.863	−0.441	0.395	
5		3	7	0.363	0.227	−1.288	0.600	−0.671	0.312	
6	BMA & MMA	7	3	1.569	0.500	−1.569	4.922	−0.222	0.697	2.141
7		6	4	1.008	0.332	−2.047	3.082	−0.392	0.590	
8		5	5	0.672	0.225	−2.384	2.055	−0.568	0.490	
9		4	6	0.448	0.143	−2.753	1.435	−0.770	0.401	
10		3	7	0.288	0.089	−2.949	0.933	−0.959	0.303	
11	EMA & BMA	7	3	2.941	2.572	1.797	3.366	0.381	0.714	1.349
12		6	4	1.891	1.714	0.785	2.091	0.228	0.608	
13		5	5	1.260	1.171	0.183	1.358	0.068	0.501	
14		4	6	0.840	0.762	−0.265	0.929	−0.116	0.408	
15		3	7	0.540	0.547	−0.460	0.540	−0.243	0.286	

Similarly, –OCH_2_– and –OCH_3_ resonances did not split while varying the composition of copolymer PMMA-*c*-PBMA. The sharp peaks at 3.96 (c) and 3.58 (a) could still determine the composition of copolymer samples. While the *α* was calculated as 2.141, the linear regression equation is ‘*η* = 1.90*ε* − 1.52’ (Fig. 7) and the intercepts at *ε* = 0 and *ε* = 1 of the *ε versus η* plot gives *r*_BMA_ = 0.38 and *r*_MMA_ = 3.26, respectively.

**Fig. 8:**
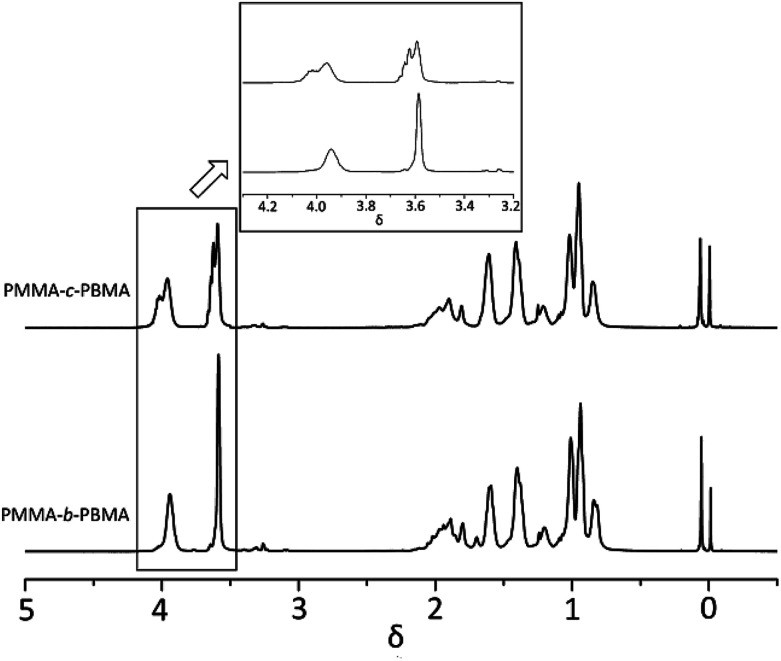
^1^H-NMR spectrum of PMMA-*c*-PBMA & PMMA-*b*-PBMA.

At this moment, it is important to point out that such a difference in the monomer reactivity ratio (0.38 : 3.26) might indicate that the early polymer must be close to PMMA in character and the later be virtually PBMA, so the opportunity for long chain segment or little block polymer would occur in the intermediate region and to a relatively small extent.^33^ When ^1^H-NMR of PMMA-*b*-PBMA and PMMA-*c*-PBMA at 30% conversions were compared in Fig. 8, it is obvious that *δ* 3.96 (c) and *δ* 3.58 (a) are the signals attributed to the PMMA and PBMA blocks. It is confirmed that little block polymer occurred in the random polymers. Additionally, the multiplet peaks around main peak were caused by different chemical environment among the block chains. This result proved the reactivity ratio corrected in one way.

However, there were overlaps between peaks at *δ* 4.02 (b) and *δ* 3.96 (c) and it was hard to vary the composition of copolymer PEMA-*c*-PBMA. Fortunately, the signals at *δ* 1.25 (d) and *δ* 1.60 (e) belonged to –CH_2_– in side chains could be used to determine the composition of copolymer samples. While the *α* was calculated as 1.349, the linear regression equation is ‘*η* = 1.56*ε* − 0.69’ (Fig. 7) and the intercepts at *ε* = 0 and *ε* = 1 of the *ε versus η* plot gives *r*_EMA_ = 0.87 and *r*_BMA_ = 0.93, respectively. It is natural that reactivity ratio was closed for the similar polarity and structures.

## Conclusions

In summary, the anionic polymerization of well-defined alkyl methacrylate block copolymers such as PMMA-*b*-PEMA, PMMA-*b*-PBMA and PEMA-*b*-PBMA was conducted with *t*-BuOK as the initiator in THF solvent, and the reaction temperature was at 0 °C without any side reaction inhibitors. The polymerization was almost completed, indicating the side reaction of anionic polymerization of alkyl methacrylate have been controlled. Specially, GPC data and ^1^H-NMR spectra revealed that the MWD of the block copolymer was rather narrow and *M*_w_ of each blocks were approximate to the calculated values. On the other hand, FTIR spectra indicated the typical symbols of blocks in copolymers and few initiator remaining in product. Furthermore, determined by DMA, the micro-phase separation clearly existed in synthesized block copolymers due to the two *T*_g_s appeared in the DMA curves. Besides, reactivity ratio were also characterized and measured, which proved that the reactivity ratio of MMA was larger than these of EMA and BMA. Accordingly, this new method with appropriate temperatures, simple initiator, fewer side reactions and a definite structure provides the possibility to realize the anionic polymerization of block copolymers containing alkyl methacrylate blocks at a commercial scale.

## Conflicts of interest

There are no conflicts to declare.

## Supplementary Material
